# Impaired phagocytic function in CX3CR1^+^ tissue‐resident skeletal muscle macrophages prevents muscle recovery after influenza A virus‐induced pneumonia in old mice

**DOI:** 10.1111/acel.13180

**Published:** 2020-07-28

**Authors:** Constance E. Runyan, Lynn C. Welch, Emilia Lecuona, Masahiko Shigemura, Luciano Amarelle, Hiam Abdala‐Valencia, Nikita Joshi, Ziyan Lu, Kiwon Nam, Nikolay S. Markov, Alexandra C. McQuattie‐Pimentel, Raul Piseaux‐Aillon, Yuliya Politanska, Lango Sichizya, Satoshi Watanabe, Kinola J.N. Williams, G. R. Scott Budinger, Jacob I. Sznajder, Alexander V. Misharin

**Affiliations:** ^1^ Division of Pulmonary and Critical Care Medicine Feinberg School of Medicine Northwestern University Chicago IL USA

**Keywords:** influenza, macrophages, pneumonia, skeletal muscle

## Abstract

Skeletal muscle dysfunction in survivors of pneumonia disproportionately affects older individuals in whom it causes substantial morbidity. We found that skeletal muscle recovery was impaired in old compared with young mice after influenza A virus‐induced pneumonia. In young mice, recovery of muscle loss was associated with expansion of tissue‐resident skeletal muscle macrophages and downregulation of MHC II expression, followed by a proliferation of muscle satellite cells. These findings were absent in old mice and in mice deficient in *Cx3cr1*. Transcriptomic profiling of tissue‐resident skeletal muscle macrophages from old compared with young mice showed downregulation of pathways associated with phagocytosis and proteostasis, and persistent upregulation of inflammatory pathways. Consistently, skeletal muscle macrophages from old mice failed to downregulate MHCII expression during recovery from influenza A virus‐induced pneumonia and showed impaired phagocytic function *in vitro*. Like old animals, mice deficient in the phagocytic receptor *Mertk* showed no macrophage expansion, MHCII downregulation, or satellite cell proliferation and failed to recover skeletal muscle function after influenza A pneumonia. Our data suggest that a loss of phagocytic function in a CX3CR1^+^ tissue‐resident skeletal muscle macrophage population in old mice precludes satellite cell proliferation and recovery of skeletal muscle function after influenza A pneumonia.

## Graphical abstract

A schematic illustrating how impaired phagocytic function in CX3CR1+ tissue‐resident skeletal muscle macrophages prevents muscle recovery after influenza A virus‐induced pneumonia in old mice

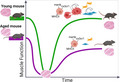

## 1 INTRODUCTION

As is evident from the ongoing coronavirus disease 2019 (COVID‐19) pandemic, elderly individuals are at increased risk of developing and dying from pneumonia (Zhou et al., 2020). Nevertheless, most elderly patients with access to modern healthcare survive (Jain et al., 2015; Thannickal et al., 2015). Elderly pneumonia survivors are at increased risk of developing age‐related disorders including persistent lung dysfunction (Mittl et al., 1994), skeletal muscle atrophy‐limiting mobility (Herridge et al., 2003), myocardial infarction (Corrales‐Medina Vicente et al., 2012), chronic kidney disease (Murugan et al., 2010), dementia (Tate et al., 2014), and cognitive impairment (Girard et al., 2018), raising significant concerns about these sequelae in elderly survivors of COVID‐19. In particular, skeletal muscle dysfunction develops in the majority of survivors of pneumonia and may persist for years after hospital discharge, where it is a major driver of morbidity (Chan et al., 2018; Dos Santos et al., 2016; Herridge et al., 2003, 2011; Walsh, Batt, Herridge, & Dos Santos, 2014). Persistent skeletal muscle weakness in survivors of severe pneumonia disproportionately affects elderly patients, resulting in reduced quality of life including an increased risk of hospitalizations, long‐term disability, and loss of independence (Barreiro, Sznajder, Nader, & Budinger, 2015; Falsey, Hennessey, Formica, Cox, & Walsh, 2005; Gozalo, Pop‐Vicas, Feng, Gravenstein, & Mor, 2012; Herridge et al., 2011; Pfoh et al., 2016).

Infection with the influenza A virus is among the most common causes of pneumonia, and like pneumonia induced by the severe acute respiratory syndrome–coronavirus‐2 (SARS‐CoV‐2), the mortality attributable to influenza A pneumonia disproportionately impacts the elderly (Jain et al., 2015; Kanegai et al., 2016; Ortiz et al., 2013; Zhou et al., 2020). Influenza A virus‐induced pneumonia in mice is an attractive model to study the effects of aging on pneumonia‐induced muscle function as it recapitulates the disproportionate skeletal muscle dysfunction reported in older survivors of pneumonia (Bartley et al., 2016). Furthermore, as the influenza A virus is trophic for the lung epithelium, there is little if any viremia (Radigan et al., 2019). In previous work foundational to the current study, we demonstrated that influenza A infection induces the expression of the muscle‐specific E3 ligase FBXO32 (atrogin‐1) and that *Fbxo32* ^−/−^ mice were resistant to influenza A‐induced muscle loss. Furthermore, we found that the administration of tocilizumab, an antibody targeting the IL‐6 receptor, to mice, prevented the induction of atrogin‐1 and the loss of muscle function after influenza A infection without affecting the severity of the lung injury or viral clearance. We concluded that the release of IL‐6 and possibly other endocrine signals from the injured lung induce the active degradation of skeletal muscle during acute influenza A‐induced pneumonia. Notably, tocilizumab and sarilumab are being tested to treat organ injury induced by the cytokine storm in patients with COVID‐19 pneumonia (Di Giambenedetto et al., 2020; Lu, Chen, & Chang, 2020).

Because skeletal muscle fibers are post‐mitotic, muscle myofibers lost during injury must be replaced through activation, proliferation, and differentiation of muscle satellite cells, a muscle‐specific progenitor cell population (Brack & Rando, 2012; Wang & Rudnicki, 2011). After injury, muscle satellite cells undergo rapid proliferation, expand in number, and fuse with damaged myofibers, restoring the number of myonuclei, myofiber content, and volume (Munoz‐Canoves & Michele, 2013; Sala et al., 2019; Tierney et al., 2014). The process of muscle repair has been studied in models of direct injury, for example, freezing, trauma, and toxin injection, but the mechanisms by which muscle fibers are regenerated after pneumonia are not known.

Given this background, the disproportionate loss of skeletal muscle mass in older pneumonia survivors could be attributable to enhanced muscle loss during the acute infection, impaired recovery, or both. We report that when the dose of influenza A administered to young and old mice is titrated to induce equivalent mortality, old and young mice suffer a nearly proportionate loss of skeletal muscle size and function. However, while young mice fully recovered muscle function to pre‐pneumonia levels 60 days after injury, old mice did not. In young mice, a population of CX3CR1‐expressing tissue‐resident macrophages in the skeletal muscle expanded and downregulated MHCII expression after influenza A infection in the absence of recruitment of monocytes from the bone marrow. This was followed by proliferation of muscle satellite cells. All of these findings were absent in old mice and mice deficient in *Cx3cr1*. Transcriptomic profiling suggested a loss of phagocytic function in tissue‐resident macrophages from the skeletal muscle of old mice, which was confirmed using in vitro assays. Knockout of *Mertk*, a tyrosine kinase involved in macrophage phagocytosis, phenocopied the molecular and physiologic findings in old mice. Our findings suggest phagocytosis‐induced signaling in CX3CR1^+^ tissue‐resident skeletal muscle macrophages is necessary for satellite cell proliferation during muscle recovery after influenza A virus‐induced pneumonia.

## 2 RESULTS

### 2.1 Old mice do not recover skeletal muscle function after influenza A‐induced pneumonia

**Figure 1:**
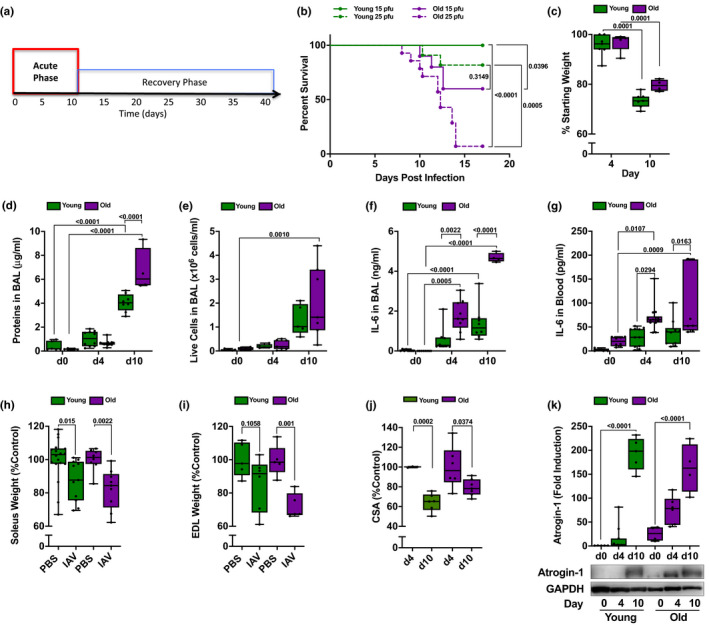
Acute influenza A virus (IAV) infection is worse in old compared with young mice. (a) Timeline of influenza A‐induced pneumonia in mice. Data in this figure focus on the acute phase (red). (b) Young (4 months old; green lines) and old (20–24 months old; purple lines) mice were infected with either 15 pfu (solid lines) or 25 pfu (dotted lines) of IAV (A/WSN/33). A Kaplan–Meier survival curve is shown (*n* = 9–14 mice per group). Log‐rank (Mantel–Cox) tests were performed, and p values are shown on graph. Each dot represents a single animal. For all data (c–k), young (4 months old; green bars) and old (20–24 months old; purple bars) mice were infected with IAV (25 pfu for young mice, 15 pfu for old mice). Mice were harvested for analysis at 0, 4, and 10 days post‐infection. A two‐way ANOVA with Tukey's post hoc corrections for comparison with more than three groups was used to determine statistical differences, or Student's *t* test for comparisons between two groups, *p* values are shown on graph. Box plot center lines are median, and box limits are upper and lower quartiles. Each dot represents an individual animal. (c) Percentage of starting total body weight is shown for day 4 and day 10 post‐IAV infection (*n* = 5–7 mice per group). (d) Total protein in bronchoalveolar lavage (BAL) fluid was measured by Bradford assay (*n* = 4–12 mice). (e) Number of live cells in the BAL was counted using hemocytometer, and dead cells were excluded using trypan blue (*n* = 5–7 mice). (f) IL‐6 levels in the BAL were measured by ELISA (*n* = 5–7 mice). (g) IL‐6 levels in the serum were measured by ELISA (*n* = 4–8 mice). (h) Soleus muscles were excised, and soleus wet muscle weights were determined. Values are presented as a percent of naive age‐matched controls (*n* = 8–18 mice). (i) Extensor digitorum longus (EDL) muscles were excised, and EDL wet muscle weights were measured. Values are presented as a percent of naive age‐matched controls (*n* = 5–7 mice). (j) Soleus cross‐sectional areas were determined using transverse cross sections immunostained for laminin. Values are normalized to naive age‐matched controls (*n* = 4–7 mice). (k) Quantification (top panel) and representative Western blot (bottom panel) of atrogin‐1 expression in the mouse tibia anterior muscle. Values are normalized to GAPDH (loading control) (*n* = 5–11 mice)

In mice, the course of influenza A follows a predictable and consistent pattern in which replicating viruses are detectable by plaque assay over the first 2–5 days of the infection accompanied by the recruitment of neutrophils and monocyte‐derived alveolar macrophages (Peteranderl et al., 2016). Mice then develop progressive hypoxemia, respiratory failure, and death (Traylor, Aeffner, & Davis, 2013), or begin a long process of lung repair, which is incomplete even months after the injury (Kanegai et al., 2016) (Figure 1a). During the acute phase of viral replication, blood cytokines including IL‐6 are dramatically increased coinciding with the loss of muscle mass. We reported that the IL‐6‐dependent activation of FBXO32 in the skeletal muscle was necessary for the acute loss of muscle mass during IAV infection (Radigan et al., 2019).

Critically ill elderly patients with pneumonia have been reported to have both enhanced muscle loss during their ICU stay and prolonged muscle dysfunction that persists more than a year after injury. Modeling this in mice poses challenges, as the LD50 for influenza A virus is dramatically different in old mice compared with young mice (Figure 1b). Accordingly, we developed a model in which we measured muscle loss and recovery in young and old mice treated with a dose of virus that resulted in similar (~25%) mortality and weight loss in each group (25 pfu per mouse for young mice and 15 pfu per mouse for old mice, Figure 1b,c). Despite the reduced dose of virus, old mice still had worse lung injury as measured by bronchoalveolar lavage protein content 10 days after infection (Figure 1d, e) and increased levels of IL‐6 in bronchoalveolar lavage fluid (Figure 1f) and in the plasma (Figure 1g) compared with young mice. The moderate age‐related increase in systemic inflammation corresponds with injury to the hind limb muscles that is only slightly worse in old compared with young mice. The soleus muscle wet weight decreased similarly in both young and old mice (Figure 1h), but the loss of wet weight in the extensor digitorum longus (EDL) muscle was more pronounced in old mice (Figure 1i). In the soleus muscle, the average fiber cross‐sectional area (CSA) was significantly reduced in both young and old mice after influenza A virus‐induced pneumonia (Figure 1j), although the proportional reductions were smaller in old mice due to a lower starting volume (Figure S1). Consistent with the higher levels of plasma IL‐6, the increase in skeletal muscle atrogin‐1 occurred more rapidly in old animals, but was similar 10 days after infection in young and old mice (Figure 1k).

**Figure 2:**
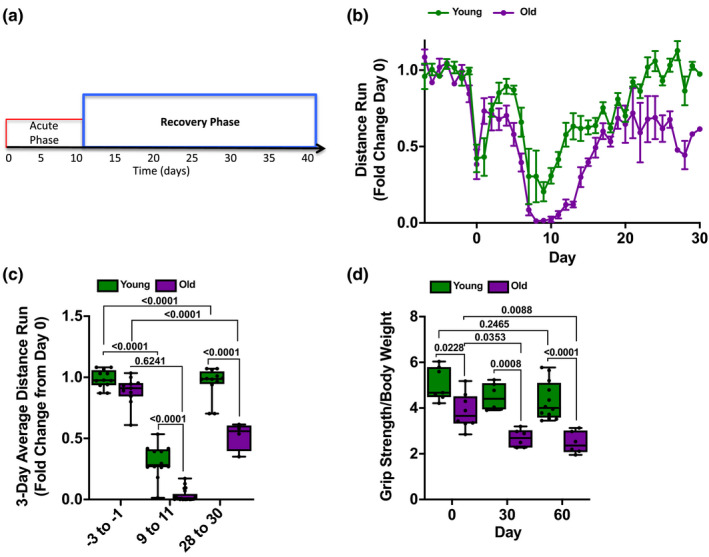
Old mice fail to recover skeletal muscle function after influenza A infection. (a) Timeline of influenza A‐induced pneumonia in mice. Data in this figure focus on the recovery phase (blue). (b) Monitored running wheels were placed in cages, and the voluntary distance run was measured for 14 days before animals were infected with influenza A virus (IAV). At day 0, young (4 months old; green lines) mice were infected with 25 pfu of IAV and old (20–24 months old; purple lines) mice were infected with 15 pfu IAV. Voluntary wheel running was recorded for 30 days, and the 8 p.m. to 6 a.m. average wheel rotation/mouse, normalized to the 10‐day average control, is shown (*n* = 12 mice per group). A graph of the distance run is shown with means ± *SD* See also Figure S2. (c) Three‐day average voluntary wheel running distance per cage normalized to the 10‐day average control is shown for young mice (4 months old; green bars) and old mice (20–24 months old; purple bars). The average distance run for days −3 to −1 prior to infection with IAV (25 pfu for young mice, 15 pfu for old mice), days 9–11 post‐infection, and days 28–30 post‐infection is shown (*n* = 12 mice per group). Box plot center lines are median, box limits are upper and lower quartiles, whiskers are minimal and maximal values, a two‐way ANOVA with Tukey's post hoc corrections for comparison with more than three groups was used to determine statistical differences, and p values are shown on graph. Each dot represents a single animal. (d) Young (4 months old; green bars) and old (20–24 months old; purple bars) mice were infected with IAV at day 0 (25 pfu for young mice, 15 pfu for old mice). Forelimb grip strength was measured at the indicated times (*n* = 6–12 mice per group). Box plot center lines are median, box limits are upper and lower quartiles, whiskers are minimal and maximal values, a two‐way ANOVA with Tukey's post hoc corrections for comparison with more than three groups was used to determine statistical differences, and p values are shown on graph. Each dot represents a single animal

As a physiologic measure of skeletal muscle function (Figure 2a), we continuously recorded voluntary running distance on monitored exercise wheels in young and old mice over the course of influenza A infection and recovery. Mice adapted to the wheels over 14 days, after which the voluntary distance run on the wheel was consistent and similar between animals (Figure 2b), although on average it was lower in old mice (5.2 ± 1.1 and 2.1 ± 1.2 km/night/mouse in old young and old mice, respectively, Figure S2a). Once baseline running distances were established, mice were infected with influenza A virus and then monitored continuously for up to 60 days. Both young and old mice almost stopped running after the influenza A virus infection, reaching a minimum at day 10 post‐infection (Figure 2b, c). However, by day 20, the distance run returned to pre‐infection levels in young mice. In contrast, old mice failed to return to their pre‐infection activity levels (Figure 2b, c). Similarly, while influenza A virus pneumonia induced a loss of forelimb grip strength in both young and old mice during the acute phase of infection (Figure S2b), old mice failed to recover forelimb grip strength 30 and 60 days after influenza A virus infection (Figure 2d). Hence, when young and old mice are infected with doses of influenza A virus titrated to result in significant but similar weight loss and mortality, old mice have only slightly worse muscle loss. However, while young mice completely recover, old mice do not.

### 2.2 Satellite cells and fibroadipogenic progenitors fail to expand in old mice during recovery from influenza A virus‐induced pneumonia

**Figure 3:**
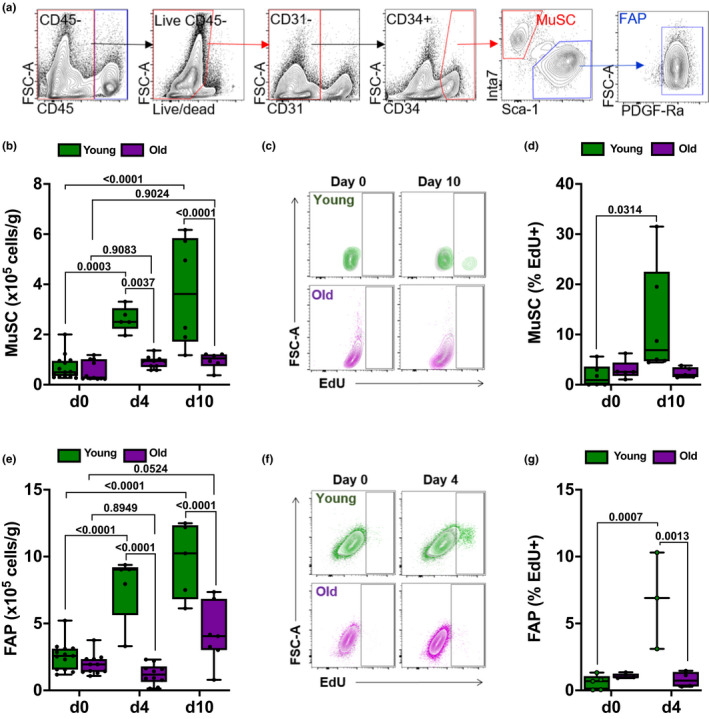
Expansion of muscle satellite cells and fibroadipogenic progenitors is reduced in old compared with young mice after influenza A virus (IAV) pneumonia. For all experiments, young (4 months old; green bars) and old (20–24 months old; purple bars) mice were infected with IAV (25 pfu for young mice, 15 pfu for old mice). Mice were harvested for analysis before the infection (day 0) and 4 and 10 days after infection. A two‐way ANOVA with Tukey's post hoc corrections for comparison with more than three groups was used to show statistical differences. Box plot center lines are median, box limits are upper and lower quartiles, whiskers are minimal and maximal values, p values are shown on the graph. Each dot represents an individual animal. (a) Representative gating strategy to identify muscle satellite cells (MuSCs) and fibroadipogenic progenitors (FAPs). (b) Enumeration of MuSC using flow cytometry. Values shown are per gram of tissue (*n* = 6–11 mice). (c) Young (top 2 panels; green) or old mice (bottom 2 panels; purple) were injected with 2 mg EdU, 16 hr prior to day 0 or day 10. Representative contour plots showing percentage of EdU‐positive MuSC are shown. Plots from EdU‐injected mice are overlaid against plots of a separate, non‐EdU‐injected control mouse (shown in gray). (d) Box and whiskers plot showing percent EdU‐positive MuSC of total MuSC at days 0 and 10 (*n* = 5–6 mice). (e) Enumeration of FAP using flow cytometry. Values shown are per gram of tissue (*n* = 5–13 mice). (f) Young (top 2 panels; green) or old mice (bottom 2 panels; purple) were injected with 2 mg EdU 16 hr prior to day 0 or day 10. Representative contour plots showing percentage of EdU‐positive FAP are shown. Plots from EdU‐injected mice are overlaid against plots of a separate, non‐EdU‐injected control mouse (shown in gray). (g) Box and whiskers plot showing percent EdU‐positive FAP of total FAP at day 0 and day 4 (*n* = 3–5 mice)

We questioned whether the failure to recover muscle strength and function in old mice was due to a failure to activate regenerative cell populations. We used flow cytometry to simultaneously quantify muscle satellite cells (MuSCs) and fibroadipogenic progenitors (FAPs) as well as lymphoid and myeloid immune cells during muscle recovery after influenza A‐induced lung injury. We identified MuSC as CD45^–^CD31^–^CD34^+^Sca‐1^–^Itga7^+^ and FAP as CD45^–^CD31^–^CD34^+^Sca‐1^+^Itga7^+^ (Figure 3a). In young, but not in old mice, we observed an expansion of MuSC and FAP after influenza A virus‐induced pneumonia (Figure 3b‐G). This suggests that muscle dysfunction induced by influenza A pneumonia is sufficient to induce regenerative cell populations in young mice, but that this response is impaired in old animals.

### 2.3 Tissue‐resident CX3CR1+ skeletal muscle macrophages expand and downregulate MHCII during recovery from influenza A virus‐induced pneumonia

**Figure 4:**
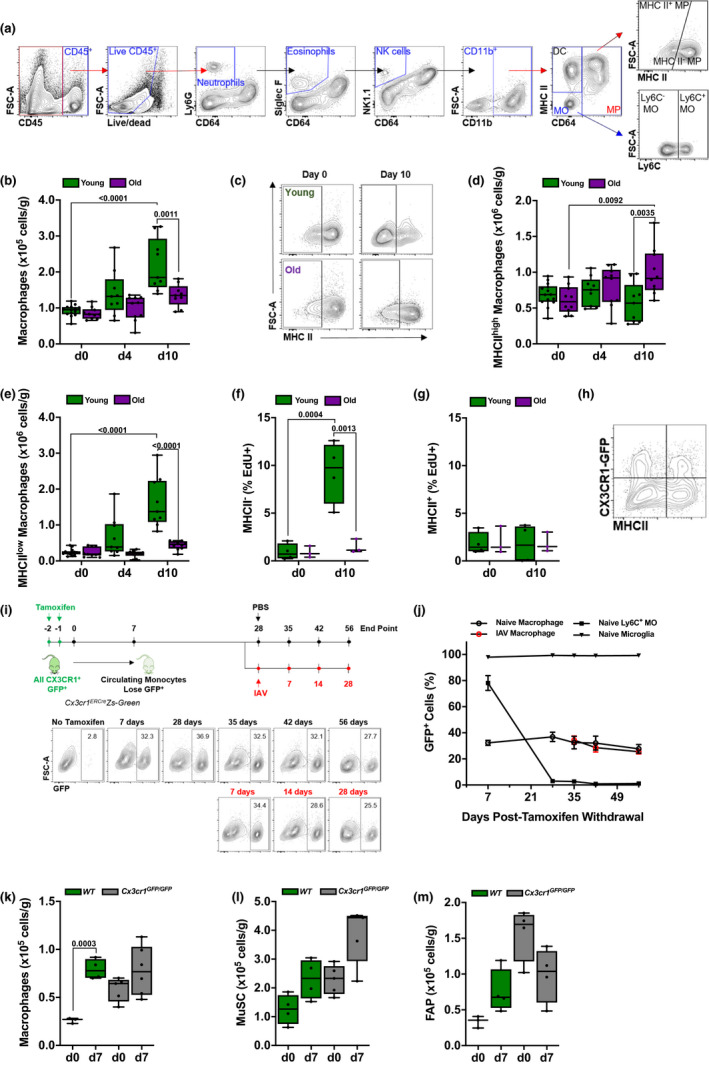
Tissue‐resident skeletal muscle macrophages expand and downregulate MHCII expression after influenza A pneumonia in young, but not in old mice. (a) Gating strategy to identify myeloid cell populations in skeletal muscle tissue using flow cytometry. (b) Young (4 months old; green bars) and old (20–24 months old; purple bars) mice were infected with influenza A virus (IAV) (25 pfu for young mice, 15 pfu for old mice). Graph shows number of macrophages per gram of tissue, before infection (day 0) and 4 and 10 days post‐infection (*n* = 9–14 mice). (c) Representative flow cytometry showing MHCII staining in skeletal muscle macrophages from young (top two panels) or old (bottom two panels) mice before and 10 days after IAV infection. (d) Young (4 months old; green bars) and old (20–24 months old; purple bars) mice were infected with IAV (25 pfu for young mice, 15 pfu for old mice). Graph shows number of MHCII^high^ macrophages in skeletal muscle per gram of tissue before infection (day 0) and 4 and 10 days after influenza A infection (*n* = 9–14 mice). (e) Young (4 months old; green bars) and old (20–24 months old; purple bars) mice were infected with IAV (25 pfu for young mice, 15 pfu for old mice). Graph shows number of MHCII^low^ macrophages in skeletal muscle per gram of tissue before infection (day 0) and 4 and 10 days after influenza A infection (*n* = 9–14 mice). (f) Young (4 months old; green bars) and old (20–24 months old; purple bars) mice were infected with IAV (25 pfu for young mice, 15 pfu for old mice). Graph shows percentage of EdU‐positive MHCII^high^ macrophages, of total MHCII^high^ macrophages, in skeletal muscle before infection (day 0) and 10 days after influenza A infection (*n* = 3–4 mice). (g) Young (4 months old; green bars) and old (20–24 months old; purple bars) mice were infected with IAV (25 pfu for young mice, 15 pfu for old mice). Graph shows percentage of EdU‐positive MHCII^low^ macrophages, of total MHCII^low^ macrophages, in skeletal muscle before infection (day 0) and 10 days after influenza A infection (*n* = 3–4 mice). (h) Representative plot of macrophages from *Cx3cr1^Gfp/WT^* mice showing heterogeneous expression of MHCII and CX3CR1 (GFP). (i) *Cx3cr1^ERCre^/zsGreen* mice were administered tamoxifen (10 mg via oral gavage, twice, 24 hr apart). Mice were harvested 7, 28, 35, 42, and 56 days after the tamoxifen pulse. A separate cohort of mice was infected with 25 pfu IAV 28 days after the tamoxifen pulse and harvested 7, 14, and 28 days later. Representative flow cytometry plots of macrophage GFP expression at each of these time points are shown. (j) Refers to the schematic in (i). *Cx3cr1^ERCre^/zsGreen* mice were administered a pulse of tamoxifen (10 mg via oral gavage, twice, 24 hr apart) at day 0. The percentage of GFP^+^ cells from the indicated cell populations is shown. Some mice were infected with 25 pfu IAV 28 days after the tamoxifen pulse and harvested 7, 14, and 28 days later. Black symbols indicate naïve mice. Red symbols indicate IAV‐infected mice. (k) Young wild‐type mice (4 months old; green bars) and age‐matched *Cx3cr1^Gfp/Gfp^* mice (gray bars) were infected with 25 pfu of IAV. Graph represents skeletal muscle macrophage number before infection (day 0) and 7 days after influenza A infection. Values are expressed per gram of tissue (*n* = 4–5 mice). (l) Young wild‐type mice (4 months old; green bars) and age‐matched *Cx3cr1^Gfp/Gfp^* mice (gray bars) were infected with 25 pfu of IAV. Graph represents muscle satellite cell (MuSC) number before infection (day 0) and 7 days after influenza A infection. Values are expressed per gram of tissue (*n* = 4–5 mice). (m) Young wild‐type mice (4 months old; green bars) and age‐matched *Cx3cr1^Gfp/Gfp^* mice (gray bars) were infected with 25 pfu of IAV. Graph represents fibroadipogenic progenitor (FAP) number before infection (day 0) and 7 days after influenza A infection. Values are expressed per gram of tissue (*n* = 4–5 mice). For all experiments, a two‐way ANOVA with Tukey's post hoc corrections for comparison with more than three groups was used to show statistical differences, and p values are shown on the graph. Each dot represents an individual animal. Also, see Figure S4

We measured myeloid and lymphoid populations in the skeletal muscle after influenza A infection using flow cytometry (Figures 4a and S3m). In young mice, we observed increases in the number of skeletal muscle macrophages (CD45+Ly6G‐Siglec‐F‐NK1.1‐CD11b+Ly6C‐CD64+) after influenza A infection (Figure 4b). This increase in skeletal muscle macrophages was not observed in old mice (Figure 4b). In contrast, we did not observe influenza A virus‐induced increases in the numbers of neutrophils, eosinophils, natural killer cells, CD11b+ dendritic cells, Ly6C‐high classical monocytes or Ly6C‐low non‐classical monocytes, CD4 and CD8 T cells, regulatory T cells, or B cells in the skeletal muscle of either young or old mice after influenza A infection (Figure S3).

As we observed a significant expansion in macrophages after influenza A infection in young mice, we focused our attention on these cells. In naïve mice, the majority of macrophages were represented by an MHCII^high^ population (Figure 4c). During recovery from influenza A infection, the majority of macrophages were MHCII^low^ in young, but not in old mice (Figure 4c‐e). Furthermore, incorporation of EdU into MHCII^low^ macrophages in after pneumonia‐induced muscle atrophy increased in young but not old mice, indicating an age‐dependent proliferation specific to this population (Figure 4f,g). Using reporter mice in which GFP is knocked into the Cx3cr1 gene (*Cx3cr1^GFP/+^*), we found that approximately 25%–30% of skeletal muscle macrophages expressed *Cx3cr1* in naïve mice (Figure S4), and both CX3CR1^+^ and CX3CR1^‐^ macrophages included populations that were MHCII^high^ and MHCII^low^ (Figure 4h).

To determine whether these skeletal muscle macrophage populations are maintained by local proliferation or are replenished by monocytes recruited from the circulation, we performed fate‐mapping experiments using *Cx3cr1^ER‐Cre^* mice crossed to a *ZsGreen^LSL^* reporter. We administered a tamoxifen pulse (two doses of tamoxifen, one day apart via oral gavage) to *Cx3cr1^ER‐Cre^/ZsGreen^LSL^* mice to permanently label all *Cx3cr1*‐expressing cells with GFP and analyzed the number of GFP‐positive cells in several myeloid populations over the course of 56 days (Figure 4i,j). Microglia, which are tissue‐resident brain macrophages that are not replaced by circulating monocytes over the lifespan of mice and are characterized by constitutive expression of *Cx3cr1* (Yona et al., 2013), were used as a positive control. Nearly 100% of microglia were labeled and remained GFP‐positive after the tamoxifen pulse after 56 days (Figure 4j). Seven days after the tamoxifen pulse, 80% of Ly6C+ classical monocytes in the blood were GFP‐positive (Figure 4j). Consistent with the short half‐life of circulating monocytes, almost all GFP‐positive monocytes were replaced by GFP‐negative monocytes 28 days after the tamoxifen pulse (Figure 4j) (Yona et al., 2013). Similarly, 28 days after the tamoxifen pulse nearly all Ly6C‐ non‐classical monocytes, and CD11b+ dendritic cells were replaced by GFP‐negative cells (not shown). Consistent with our findings using *Cx3cr1^Gfp/WT^* mice, we found that ~ 30% of skeletal muscle macrophages were GFP‐positive after the tamoxifen pulse (Figure 4j). The number of GFP‐positive skeletal muscle macrophages remained stable 56 days after the tamoxifen pulse. As such, these cells represent a *bona fide,* long‐lived tissue‐resident macrophage population that maintain their population without input from circulating monocytes with a half‐life of at least several months.

We used the *Cx3cr1^ER‐Cre^/ZsGreen^LSL^* mice to determine whether the increase in skeletal muscle macrophages we observed after influenza A virus‐induced pneumonia was attributable to the recruitment of monocyte‐derived macrophages or the proliferation of tissue‐resident macrophages. We treated *Cx3cr1^ER‐Cre^/ZsGreen^LSL^* with two pulses of tamoxifen one day apart, waited 28 days for GFP‐labeled monocytes to disappear, infected the mice with influenza A virus, and then harvested mice 7, 14, and 28 days after infection (Figure 4i). We did not observe any reduction in the percentage of GFP‐positive macrophages, excluding the possibility that the increase in skeletal muscle macrophages following influenza A infection represented an influx of circulating cells (Figure 4i,j). Further, the finding that the number of macrophages expanded after influenza A infection combined with the constant percentage of GFP‐positive cells suggests both CX3CR1‐positive and CX3CR1‐negative cells expand proportionately after influenza A infection (Figure 4b,j). CX3CR1 is required for the adhesion and migration of leukocytes, and in the microglia, CX3CR1 is necessary for synaptic pruning during development (Paolicelli et al., 2011). We therefore sought to determine whether CX3CR1 is necessary for the expansion of macrophages and recovery of muscle function after influenza A infection. We treated mice deficient in *Cx3cr1* (*Cx3cr1^Gfp/Gfp^*) with influenza A virus and performed flow cytometry on skeletal muscle during recovery. Mice deficient in *Cx3cr1* showed increased numbers of skeletal muscle macrophages at baseline, perhaps reflecting a failure to populate the niche. However, *Cx3cr1^Gfp/Gfp^* mice showed no expansion of skeletal muscle macrophages, no change in the proportion of MHCII^low^ macrophages, and no proliferation of MuSC or FAP after influenza A virus‐induced pneumonia (Figure 4k–m). This suggests that functional expression of *Cx3cr1* on macrophages that reside in skeletal muscle is required for a proliferative response of MuSC and FAP to injury.

### 2.4 Transcriptomic profiling identifies downregulation of phagocytic and proteostatic pathways and upregulation of inflammatory pathways in tissue‐resident skeletal muscle macrophages from old compared with young mice

**Figure 5:**
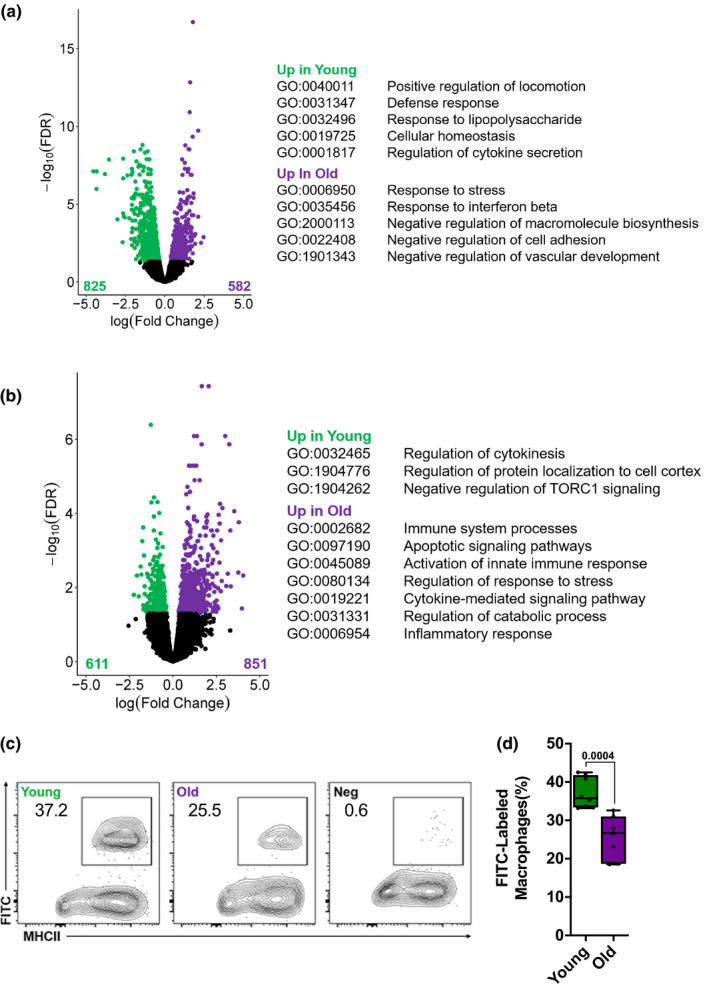
Tissue‐resident macrophages from skeletal muscle from old mice show impaired phagocytosis. (a) Skeletal muscle macrophages were flow cytometry‐sorted from naïve young (4 months old) and old (20–24 months old) mice and analyzed using RNA‐seq (*n* = 5 mice per group). Differentially expressed genes (FDR q < 0.05) between young (green dots) and old (purple dots) mice are shown with representative genes and GO biological processes (see Tables S1 and S2 for complete list of genes and GO processes). (b) Young (4 months old) and old (20–24 months old) mice were infected with influenza A virus (25 pfu for young mice and 15 pfu for old mice) and harvested 60 days later. Skeletal muscle macrophages were flow cytometry‐sorted and analyzed using RNA‐seq (*n* = 5 mice per group). Differentially expressed genes (FDR *q* < 0.05) between young (green dots) and old mice (purple dots) are shown with representative genes and GO biological processes (see Tables S3 and S4 for complete list of genes and GO processes). (c) Single‐cell suspensions prepared from skeletal muscle of young (4 months old) and old (20–24 months old) mice were incubated with antibodies to detect myeloid cells and serum‐opsonized Fluoresbrite microspheres. Uptake of the fluorescently labeled microspheres by macrophages was measured using flow cytometry. Non‐opsonized beads were used as a negative control. Representative contour plots are shown, and numbers indicate percentage from the parent population (gated on macrophages; see Figure 4a for gating strategy). (d) Refers to (c). Graph showing average percent of phagocytosed microspheres per macrophage (*n* = 7–8 mice). A two‐way ANOVA with Tukey's post hoc corrections for comparison with more than three groups was used to show statistical differences. Box plot center lines are median, box limits are upper and lower quartiles, whiskers are minimal and maximal values, and p values are shown on the graph. Each dot represents an individual animal

To identify possible mechanisms responsible for the lack of a reparative response in tissue‐resident skeletal macrophages from old mice during recovery from influenza A virus‐induced pneumonia, we performed transcriptomic profiling (RNA‐seq) of flow cytometry‐sorted skeletal muscle macrophages. Out of 1,407 differentially expressed genes (FDR *q* < 0.05), 582 were upregulated in skeletal muscle macrophages from old compared with young animals and 825 were downregulated (Figure 5a). Compared with young mice, skeletal muscle macrophages from naïve old animals demonstrated decreased expression of genes involved lipid storage and metabolism (*Ldlrap1, Lrp1, Fabp4, Nr1h3*), macrophage activation and cytokine production (*Nlrp3*, *Sphk1, Msr1, Il10*), and extracellular matrix remodeling (*Spp1, Mmp13, Mmp14, Thbs1, Chil3*) (Figure 5a and Tables S1 and S2, list of DEGs and corresponding GO processes). In addition, macrophages from old animals demonstrated decreased expression of the genes involved in response to unfolded protein and cellular stress, particularly molecular chaperones (*Dnaja2, Dnajb4, Dnajb11, Hspa9, Hsp90b1, Hspbap1*), as well as several molecules involved in positive regulation of phagocytosis (*C5ar1, Msr1*). In contrast, macrophages from young naïve animals were characterized by decreased expression of the genes involved in negative regulation of cell migration (*Sema4a, Sema6d, Plxna3, Sema4c, Sema6a*) and antigen processing and presentation (*Cd74, H2‐Ab1, H2‐Eb1, H2‐Aa*). We also performed transcriptomic profiling of flow cytometry‐sorted skeletal muscle macrophages from young and old mice 60 days after IAV pneumonia. Of 1,054 differentially expressed genes (FDR q < 0.05), 673 genes were upregulated in skeletal muscle macrophages from old mice and were enriched for the genes involved in inflammation (*Il6, Tnf, Il10, Il1a, Il1b, Tnfsf14*) and proteostatic stress (*Hspa9, Hspa5, Hspb8, Hspbap1, Dnaja4, Dnajb4, Dnaja2*) (Figure 5b and Tables S3 and S4, list of DEGs and corresponding GO processes). These transcriptomic data suggested impaired phagocytosis and increased inflammatory response in skeletal muscle macrophages from old mice. Consistent with these observations, we found that flow cytometry‐sorted tissue‐resident skeletal muscle macrophages from old mice exhibited reduced uptake of fluorescently labeled polystyrene beads when compared with those from young animals (Figure 5c, d).

### 2.5 The phagocytic receptor *Mertk* is necessary for skeletal muscle repair after influenza A‐induced pneumonia

**Figure 6:**
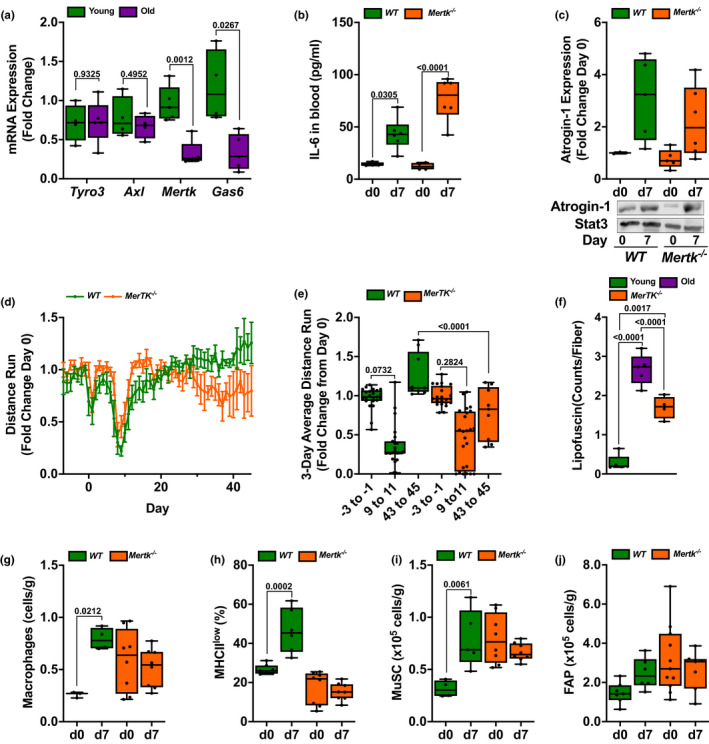
*Mertk* is necessary for recovery of skeletal muscle function after influenza A‐induced pneumonia. (a) Skeletal muscle macrophages were isolated from naïve young (4 months: green bars) and old (20–24 months: purple bars) mice, and RNA was analyzed for TAM family members via real‐time qPCR with SYBR Green (*n* = 5 mice). (b) Young (4 months old: orange bars) *Mertk* ^−/−^ mice and age‐matched wild‐type control mice (green bars) were infected with influenza A virus (IAV) (25 pfu) and harvested before the infection (day 0) and 7 days after the infection. IL‐6 was measured by ELISA in the serum (*n* = 4–6 mice). (c) Young (4 months old: orange bars) *Mertk* ^−/−^ mice and age‐matched wild‐type control mice (green bars) were infected with IAV (25 pfu) and harvested before the infection (day 0) and 7 days after the infection. Quantification (top panel) and representative Western blot (bottom panel) of atrogin‐1 expression in the mouse tibialis anterior muscle. GAPDH was used as a loading control (*n* = 3–6 mice). (d) Prior to being infected with IAV, young (4 months) *Mertk* ^−/−^ mice (orange lines) and age‐matched wild‐type control mice (green lines) were exposed to voluntary wheel running for 14 days. They were then infected with 25 pfu of IAV, and voluntary wheel running was recorded for 50 days. The 8 p.m. to 6 a.m. average wheel rotation/mouse, corrected to the initial 10‐day average control, is shown (*n* = 15 mice per group). Graph shows distance run with means ± *SD* (e) Mice were treated as in (d). The 3‐day average voluntary wheel running per cage is shown for days −3 to −1 prior to IAV, days 9–11 post‐infection, and days 43 to 45 post‐infection (*n* = 15 mice per group). (f) Graph depicting number of lipofuscin granules per fiber cross section counted manually in Image J. Five mice per group (an average of 3 fields of view per mouse). (g) Young (4 months old, orange bars) *Mertk* ^−/−^ mice and age‐matched wild‐type control mice (green bars) were infected with IAV (25 pfu) and harvested before the infection (day 0) and 7 days after the infection. Skeletal muscle macrophages were enumerated using flow cytometry (*n* = 4–8 mice). Values are expressed per gram of tissue. (h) Young (4 months old, orange bars) *Mertk* ^−/−^ mice and age‐matched wild‐type control mice (green bars) were infected with IAV (25 pfu) and harvested before the infection (day 0) and 7 days after the infection. MHCII^low^ skeletal muscle macrophages were enumerated using flow cytometry (*n* = 4–8 mice). Values are expressed as a percent of total macrophages. (i) Young (4 months old, orange bars) *Mertk* ^−/−^ mice and age‐matched wild‐type control mice (green bars) were infected with IAV (25 pfu) and harvested before the infection (day 0) and 7 days after the infection. Skeletal muscle satellite cells (MuSCs) were enumerated using flow cytometry (*n* = 4–8 mice). Values are expressed per gram of tissue. (j) Young (4 months old, orange bars) *Mertk* ^−/−^ mice and age‐matched wild‐type control mice (green bars) were infected with IAV (25 pfu) and harvested before the infection (day 0) and 7 days after the infection. Skeletal muscle fibroadipogenic progenitor (FAP) cells were enumerated using flow cytometry (*n* = 4–8 mice). Values are expressed per gram of tissue. For all experiments, a two‐way ANOVA with Tukey's post hoc corrections for comparison with more than three groups was used to show statistical differences. Box plot center lines are median, box limits are upper and lower quartiles, whiskers are minimal and maximal values, and p values are shown on the graph. Each dot represents an individual animal

Our transcriptomic data suggested impaired phagocytosis in old compared with young animals. Furthermore, the persistence of MHCII expression in skeletal muscle macrophages from old mice after influenza A infection might reflect impaired phagocytosis (Lemke, 2013). MERTK is a member of the TAM family (Tyro3/Axl/Mer) of tyrosine kinases that is expressed on macrophages, and is involved in recognizing and clearing apoptotic cells through phagocytosis (Lemke, 2013). We found that the level of mRNA encoding *Mertk* and its phosphatidylserine binding co‐receptor *Gas6* were reduced in flow cytometry‐sorted macrophages from old compared with young mice (Figure 6a). Accordingly, we infected *Mertk* ^−/−^ and wild‐type mice with influenza A virus and measured skeletal muscle injury and recovery post‐infection. Wild‐type and *Mertk* ^−/−^ mice developed comparable levels of systemic IL‐6 and atrogin‐1 expression in the skeletal muscle (Figure 6b, c), suggesting a similar level of muscle injury. However, measurements of voluntary distance run on a monitored exercise wheel showed that after an initial transient recovery, *Mertk* ^−/− ^mice failed to maintain their pre‐infection levels of activity after pneumonia (Figure 6d,e). Additionally, we found increased accumulation of age‐related autofluorescent granules representing either lipofuscin or ceroid in the muscle of young *Merkt* ^−/− ^mice compared with young wild‐type mice, and a similar accumulation of lipofuscin or ceroid was observed in old mice (Figures 6f and S5). Similar to *Cx3cr1* knockout mice, we observed increased numbers of skeletal muscle macrophages in young *Mertk* ^−/−^ mice at baseline. However, the number of skeletal muscle macrophages in young *Mertk* ^−/−^ mice did not increase during recovery from influenza A virus‐induced pneumonia (Figure 6g) and there was no increase in the proportion of macrophages that were MHCII^low^ (Figure 6h), similar to old mice. Consistent with these findings, *Mertk* ^−/− ^mice lacked expansion of MuSC and FAP during recovery from influenza A‐induced pneumonia (Figure 6i,j). These data support a requirement for functional phagocytosis in recovery from influenza A pneumonia‐induced skeletal muscle atrophy.

## 3 DISCUSSION

As vividly illustrated by the COVID‐19 pandemic, pneumonia disproportionately affects the elderly (Zhou et al., 2020). While mortality from pneumonia is higher in older compared with younger individuals, most elderly patients with access to modern healthcare survive their pneumonia (Jain et al., 2015; Ortiz et al., 2013). It is well established that skeletal muscle dysfunction is common in survivors of severe pneumonia requiring intensive care (Herridge et al., 2003). This skeletal muscle dysfunction after pneumonia is disproportionately prolonged and severe in the elderly when compared to younger individuals; however, the mechanisms that underlie this age‐related susceptibility have not been elucidated (Barreiro et al., 2015). In foundational work in advance of this study, we used a murine model of influenza A pneumonia to show the release of IL‐6 from the injured lung signaled the expression of the muscle‐specific E3 ligase FBXO32, actively inducing skeletal muscle degradation. Here, we recapitulate previous findings that old compared with young mice suffer increased mortality when infected with the same dose of influenza A virus. To compare the mechanisms of skeletal muscle dysfunction in young and old mice, we reduced the dose of virus administered to old mice to cause similar mortality and weight loss when compared with young mice. Even at this reduced dose, the levels of IL‐6 and other inflammatory cytokines were increased in old compared with young mice and the severity of acute muscle loss was worse in old compared with old mice. While the differences in acute muscle loss in old compared with young mice were subtle, the differences in recovery were striking. Young animals completely recovered skeletal muscle function after pneumonia, while old animals did not. We used a combination of genetic lineage tracing, flow cytometry, transcriptomics, and genetic loss‐of‐function approaches to show that a loss of phagocytic function in tissue‐resident CX3CR1^+^ skeletal muscle macrophages contributes to the failure of skeletal muscle recovery in old mice after influenza A infection. Our findings take on new importance during the COVID‐19 pandemic where therapies targeting the IL‐6 receptor are being widely used to target the systemic inflammatory response induced by the SARS‐CoV‐2 coronavirus (Giamarellos‐Bourboulis et al., 2020).

While a subpopulation of macrophages expressing the fractalkine receptor CX3CR1 in the skeletal muscle has been previously reported, nothing was known regarding the function or ontogeny of these cells (Arnold et al., 2015; Jin, Warunek, & Wohlfert, 2018; Zhao, Lu, Wang, Ransohoff, & Zhou, 2016). In young mice recovering from influenza A pneumonia, we observed expansion and downregulation of MHCII in skeletal muscle macrophages followed by muscle satellite cell proliferation. These findings were absent in mice deficient in *Cx3cr1*. Because macrophages are the only cell type in the skeletal muscle that expresses *Cx3cr1* (Schaum et al., 2018), these findings genetically link CX3CR1‐expressing skeletal muscle macrophages with the proliferation of muscle satellite cells (MuSCs) after influenza A‐induced pneumonia. Moreover, we used a lineage tracing system and flow cytometry to show that these skeletal muscle macrophages represent a *bona fide* tissue‐resident macrophage population maintained in the skeletal muscle via local proliferation independent of recruitment of monocytes or monocyte‐derived macrophages in the steady state and after influenza A pneumonia. Further, unlike other models of skeletal muscle injury and recovery induced by freezing or cardiotoxin administration, we found no evidence of infiltration by other myeloid cell populations or lymphoid populations in response to influenza A pneumonia. While the precise function of CX3CR1 in the skeletal muscle is not known, it may guide macrophages to areas of muscle loss induced by influenza A infection. For example, signaling through CX3CR1 guides monocytes and macrophages to areas of injury or inflammation, and during brain development, CX3CR1 is necessary to guide microglia to synapses where they are pruned from living neurons via a process referred to as trogocytosis (Paolicelli et al., 2011; Weinhard et al., 2018).

We used transcriptomic profiling to compare tissue‐resident skeletal muscle macrophages in young and old mice under naïve conditions and during recovery from influenza A infection. These data suggested the hypothesis that skeletal muscle macrophages from old mice lose phagocytic function, which was confirmed by reduced uptake of serum‐opsonized polystyrene beads in flow cytometry‐sorted skeletal muscle macrophages from old compared with young mice. MERTK is a member of the TAM family of scavenger receptors (MERTK, AXL, and TYRO3), which are activated during injury by interactions with Protein S or Gas6 bound to Annexin V on the surface of apoptotic cells (Lemke, 2013). Ligation of MERTK in macrophages results in the induction of phagocytosis through protein kinase C and Rac‐mediated regulation of the cytoskeleton and the activation of AKT, leading to cellular proliferation and survival (Lemke & Rothlin, 2008). In addition, the activation of MERTK results in its association with the type I interferon receptor (IFNAR) to induce the Jak1/Stat1‐mediated activation of suppressor of cytokine signaling 1 and suppressor of cytokine signaling 3 (SOCS1 and SOC3) (Lemke, 2013). These transcription factors inhibit inflammatory mediators and further reduce expression of MHC class II molecules on the cell surface (Dalpke, Opper, Zimmermann, & Heeg, 2001). We found that during recovery from influenza A infection, young *Mertk* ^−/−^ mice largely phenocopied old mice, in that they failed to expand tissue‐resident macrophages, downregulate MHCII on tissue‐resident macrophages, or expand MuSC and they failed to muscle function after influenza A infection. Our findings are consistent with previous reports that MERTK is necessary for cardiac repair after myocardial infarction and that mesenchymal stem cells promote cardiac repair by activating phagocytic pathways in cardiac macrophages, strengthening the causal association between tissue‐resident skeletal muscle macrophage function and muscle satellite cell proliferation (DeBerge et al., 2017; Vagnozzi et al., 2020; Zhang et al., 2019).

Our findings fit into an emerging body of literature implicating impaired phagocytosis in tissue‐resident macrophages with aging phenotypes. For example, Pluvinage performed a CRISPR screen to identify genes involved in microglia phagocytosis (Pluvinage et al., 2019). They found that CD22 was upregulated in aging microglia and impaired their phagocytic function. When they administered an antibody directed against CD22, cognitive function in old mice improved. Future studies will answer whether improving phagocytic function in tissue‐resident macrophages in the skeletal muscle will be sufficient to revert influenza A pneumonia‐induced muscle dysfunction in old mice. Furthermore, our results support a role for tissue‐resident macrophages in promoting cell proliferation. For example, co‐culture of monocyte/macrophage populations with muscle progenitor cells induces their proliferation via the release of soluble factors in rodent and avian systems (Cantini & Carraro, 1995; Cantini et al., 2002; Chazaud et al., 2003; Merly, Lescaudron, Rouaud, Crossin, & Gardahaut, 1999; Saclier et al., 2013). In our model, however, we cannot determine whether impaired MuSC proliferation is related to the loss of paracrine signals induced in macrophages upon uptake of cellular debris or an inhibition of cellular proliferation by the debris that is not cleared by macrophages.

Our molecular findings are consistent with the studies of muscle biopsies from patients with myopathy after critical illness. In these biopsies, investigators observed MuSC proliferation in the absence of inflammatory cell infiltration (Dos Santos et al., 2016). This differs from many murine models used to study muscle recovery, most of which involve direct injury to the muscle including trauma, freezing, or cardiotoxin injection. In all of these injuries, inflammatory cells are recruited to the injured muscle including neutrophils, monocytes, monocyte‐derived macrophages, regulatory T cells (Tregs), and other T‐cell populations (Arnold et al., 2007; Heredia et al., 2013; Kuswanto et al., 2016; Teixeira et al., 2003; Varga et al., 2016; J. Zhang et al., 2014). Inflammatory cell recruitment in those models precluded the specific study of tissue‐resident skeletal muscle macrophages. For example, two independent groups reported that deletion of monocyte‐derived macrophages recruited in response to cardiotoxin‐induced injury resulted in impaired satellite cell proliferation. In both of these studies, the strategies used to deplete monocyte‐derived macrophages (CD11b‐DTR and an antibody to M‐CSF) would target both monocyte‐derived macrophages and the skeletal muscle tissue‐resident macrophages we identified in this study (Arnold et al., 2007; Segawa et al., 2008). While Tregs have been reported to be important for skeletal muscle recovery after cardiotoxin injury (Kuswanto et al., 2016), we found that Treg number was reduced in both young and old mice after influenza A infection. Nevertheless, Treg proliferation was higher in young compared with old mice and it is possible that factors released from resident tissue Tregs are important for recovery.

Our study has some limitations. First, while voluntary wheel running in mice is an intuitive and compelling measure of the skeletal muscle dysfunction that impairs mobility in survivors of pneumonia, it is a composite measure that may be altered by factors other than muscle function. Second, while *Mertk* ^−/−^ mice recapitulated the lack of cellular responses in old animals, they transiently regained muscle function after injury. This may be explained by the high basal levels of MuSC in *Mertk* ^−/−^ relative to wild‐type mice, which allows short term, but unsustainable recovery of muscle function. Third, while we observed expansion of FAPs during recovery from muscle injury in young, old, and *Mertk*‐deficient mice, their role in muscle recovery is not certain. FAP cells have been described as important facilitators of MuSC behaviors, supporting their myogenic differentiation (Fiore et al., 2016), yet FAP cell persistence has been shown to have a detrimental effect on muscle tissue by contributing to fibrosis (Gonzalez et al., 2017) and ectopic fat accumulation (Uezumi, Fukada, Yamamoto, Takeda, & Tsuchida, 2010). Thus, if tissue‐resident macrophages contribute to FAP clearance, as has been reported for inflammatory, monocyte‐derived macrophages (Lemos et al., 2015) some of the effects of aging or *Mertk* loss in macrophages might be attributed to the persistence of these cells. Finally, while the expression of *Cx3cr1* in the skeletal muscle is limited to macrophages, *Cx3cr1* is also expressed on monocytes and LYVE1‐expressing macrophages associated with the vasculature and nerve fibers (Lim et al., 2018; Schaum et al., 2018). We therefore cannot completely exclude off target effects related to global deletion of *Cx3cr1* in our system.

Skeletal muscle represents approximately 40% of the total body mass (Janssen, Heymsfield, Wang, & Ross, 2000), and maintaining its function is critical for posture, breathing, locomotion, nutrient storage, and overall well‐being. As a result, loss of muscle function and mass with age‐related sarcopenia is a major driver of frailty (Janssen et al., 2000; Topinkova, 2008). Our findings highlight the importance of impaired repair in the persistent, sometimes life‐long loss of muscle function that develops in elderly survivors of pneumonia. Specifically, we suggest that a loss of phagocytic function in tissue‐resident skeletal muscle macrophages during aging impairs their ability to promote muscle satellite cell proliferation necessary for skeletal muscle recovery. Given the prevalence of influenza A, COVID‐19, and other respiratory viral infections, it is likely that our findings have broad implications for elderly survivors of pneumonia.

**Table:** 

Reagent or resource	Source	Identifier
Antibodies		
CD45 clone 30‐F11 PE‐Cy7	BD Biosciences	552848; RRID: 5114808
CD31 clone MEC 13.3 PE‐CF594	BD Biosciences	563616; RRID: 5163928
CD34 clone RAM34 FITC	BD Biosciences	553733; RRID: 6019764
CD34 clone RAM34 Alexa Fluor 700	eBiosciences	56‐0341‐82; RRID: 4289831
Sca‐1 clone D7 BV421	BioLegend	108128; RRID: B254500
Int‐a7 clone 3C12 APC	Miltenyi	130‐102‐717; RRID: 5190509033
PDGF‐Ra clone APA5 PE	BioLegend	135906; RRID: B203475
CD64 clone X54‐5/7.1 PE	BioLegend	139304; RRID: B191540
Ly6G clone 1A8 Alexa Fluor 700	BD Biosciences	561236; RRID: 6252780
Siglec‐F clone E50‐2440 PE‐CF594	BD Biosciences	562757; RRID: 7128860
NK1.1 clone PK136 BUV395	BD Biosciences	564144; RRID: 8155628
CD11b clone M1/70 FITC	BD Biosciences	561688; RRID: 5357544
CD11b clone M1/70 APC	BioLegend	101212; RRID: B247959
MHCII M5/114.15.2 eFluor 450	eBiosciences	48‐5321‐82; RRID: 4289694
Ly6C clone HK1.4 APC/Cy7	BioLegend	128018; RRID: B232708
CD19 clone 1D3 PE‐CF594	BD Biosciences	562329; RRID: 5336548
CD3e clone 145‐2C11 FITC	BD Biosciences	553061; RRID: E00061‐1634
CD3e clone 145‐2C11 APC/Cy7	BioLegend	100300; RRID: B239433
CD4 clone RM4‐5 eFluor 450	eBiosciences	48‐0042; RRID: 4281988
CD8 clone 53‐6.7 BUV395	BD Biosciences	563786; RRID: 7096603
CD25 clone PC61.5 APC	eBiosciences	17‐0251‐82; RRID: 4300565
FoxP3 clone FJK‐16s PE	eBiosciences	12‐5773‐82; RRID: 4279536
Fbx32 (atrogin 1)	Abcam	ab168372; RRID: GR322135−8
GAPDH	Cell Signaling	D16H11; RRID:
Laminin	Sigma	L9393; RRID: 015M4881V
Alexa Fluor 568 secondary antibody	Life Technologies	A‐11011; RRID: 2088068
Chemicals, peptides, and recombinant proteins		
AutoMACS Buffer	Miltenyi	130‐091‐221
BD Pharm Lyse	BD Biosciences	555899
eFluor 506 Viability Dye	Thermo Fisher	65‐0866‐18
123count eBeads	eBioscience	01‐1234
Anti‐mouse CD16/CD32	Invitrogen	14‐0141‐85
Fluoresbrite YG 1.0 Micron Microspheres	Polysciences, Inc	16662
RNase‐free DNase Set	Qiagen	79254
Critical commercial assays		
Skeletal Muscle Dissociation Kit	Miltenyi	130‐098‐305
Mouse IL‐6 ELISA Kit	Life Technologies	KMC0061C
FoxP3 Staining Buffer Set	eBiosciences	00‐5523
Click‐iT Plus EdU Assay Kit	Invitrogen	C10632
Arcturus PicoPure RNA Isolation Kit	Thermo Fisher	KIT0204
iScript cDNA Synthesis Kit	Bio‐Rad	1725034
iTaq SYBR One‐Step RT‐PCR kit	Bio‐Rad	1725151

**Deposited data**

**Experimental Models: Organisms/Strains**

**Table:** 

C57Bl/6 9–11 weeks/18 months	NIA NIH	
B6.129P2(Cg)‐*Cx3cr1^tm1Litt^*/J	The Jackson Laboratory	Stock #005582
B6.129P2(C)‐*Cx3cr1^tm2.1(cre/ERT2)Jung^/J*	The Jackson Laboratory	Stock #020940
B6.Cg‐*Gt(ROSA)26Sor^tm6(CAG‐ZsGreen1)Hze^/J*	The Jackson Laboratory	Stock #007906
B6;129‐*Mertk^tm1Grl^*/*J*	The Jackson Laboratory	Stock #011122

**Oligonucleotides**

**Table:** 

Gapdh FW	IDT	GCA CAG TCA AGG CCG AGA AT
Gapdh RV	IDT	GCC TTC TCC ATG GTG GTG AA
MerTK FW	IDT	CGT CTG TCC TAA CCG TAC CT
MerTK RV	IDT	GTA CTG TTG AGG ATA TGG ACT
Axl FW	IDT	GGT CCC CCT GAG AAC GTT AG
Axl RV	IDT	CAT AAG TAC CTC GGG GGT GT
Tyro3 FW	IDT	AGT GGA ACG GTC TGA TGC TG
Tyro3 RV	IDT	AGA ATG GCA CAC CTT CGA CA
Gas6 FW	IDT	ATG GGT GCA TGA GGA GTT GG
Gas6 RV	IDT	TGT TCG GGT GTA GTT GAG CC

**Software and Algorithms**

**Table:** 

FlowJo V10.1	Treestar, FlowJo, Ashland, Oregon	https://www.flowjo.com/solutions/flowjo/downloads
GraphPad Prism 7.02	GraphPad Software, California	https://www.graphpad.com/scientific-software/prism/
bcl2fastq 2.17.1.14	Illumina, California	https://support.illumina.com/sequencing/sequencing_software/bcl2fastq-conversion-software.html
Trimmomatic 0.36	Usadel Lab, RWTH Aachen University, Institute for Biology I	http://www.usadellab.org/cms/?page=trimmomatic
STAR 2.6.0	Alexander Dobin, Cold Spring Harbor Laboratory	https://github.com/alexdobin/STAR
HTSeq 0.7.1	Genome Biology Unit, EMBL Heidelberg	https://htseq.readthedocs.io/en/release_0.10.0/
edgeR 3.20.9	Bioconductor	https://bioconductor.org/packages/release/bioc/html/edgeR.html
Gorilla	Eden, Navon, Steinfeld, Lipson, Yakhini, 2009	http://cbl-gorilla.cs.technion.ac.il/
**Other**		
Dulbecco's modified Eagle's medium (DMEM)	Fisher Scientific	MT‐10‐013‐CM
2‐Mercaptoethanol	Sigma‐Aldrich	M6250
Bio‐Rad protein assay dye reagent concentrate	Bio‐Rad Laboratories	500‐0006

## 4 CONTACT FOR REAGENT AND RESOURCE SHARING

Further information and requests for resources and reagents should be directed to and will be fulfilled by the Lead Contacts: Jacob Sznajder (j‐sznajder@northwestern.edu) and Alexander Misharin (a‐misharin@northwestern.edu).

## 5 EXPERIMENTAL MODEL AND SUBJECT DETAILS

### 5.1 Mouse models

All mouse procedures were approved by the Institutional Animal Care and Use Committee at Northwestern University (Chicago, IL, USA). All strains including wild‐type mice are bred and housed at a barrier‐ and specific pathogen‐free facility at the Center for Comparative Medicine at Northwestern University. All experiments were performed with littermate controls. Number of animals per group was determined based on our previous publications. Mice were housed at the Center for Comparative Medicine at Northwestern University, in microisolator cages, with standard 12‐hr light/darkness cycle, ambient temperature 23°C, and were provided standard rodent diet (Envigo/Teklad LM‐485) and water ad libitum. Four‐ and eighteen‐month‐old C57BL/6J mice were obtained from Jackson Laboratories or NIA colony. The C57BL/6J, *Cx3cr1^ER‐Cre^* mice (Yona et al., 2013) and ZsGreen reporter (Madisen et al., 2010) mice were obtained from Jackson laboratories (Jax stocks 000664, 020940, and 007906, correspondingly) as were Cx3cr1*^gfp/gfp^* mice (Jax stock 005582).

## 6 METHODS’ DETAIL

### 6.1 Influenza A infection

Murine‐adapted, influenza A virus (A/WSN/33 [H1N1]) was provided by Robert Lamb, Ph.D., Sc.D., Northwestern University, Evanston, IL. Mice were anesthetized with isoflurane, their lungs were intubated orally with a 20‐gauge Angiocath (Franklin Lanes, NJ), and then instilled with either sterile, phosphate‐buffered saline (S), or mouse‐adapted influenza A virus (IAV) as previously described (Radigan et al., 2019). Various doses in a range of 10‐100pfu were tested to find an optimal sublethal dose matched to the age of the animal. Based on survival curves, we determined that 25 pfu was optimal for young animals, but a lower dose was required for old animals, as a 25 pfu dose resulted in excessive mortality of old mice (Figure S1). We continuously observed mice infected with influenza A virus for signs of distress (slowed respiration, failure to respond to cage tapping, failure of grooming, huddling, and fur ruffling). Mice that developed these symptoms were sacrificed, and the death was recorded as an influenza A‐induced mortality. Weight was measured prior to influenza infection and prior to harvest at 4 or 10 days post‐infection, and recorded as percentage of weight loss from baseline (Radigan et al., 2019).

### 6.2 Measurement of muscle dysfunction

Immediately prior to muscle harvest, forelimb skeletal muscle strength was assessed using a digital grip strength meter (Columbus Instruments) as described (Radigan et al., 2019). Grip strength was measured in each animal six successive times, and the average of the highest four values for each mouse was used. The same operator performed all tests. The mice were then terminally anesthetized with EUTHASOL (pentobarbital sodium/phenytoin sodium). The soleus and EDL muscles were excised, and tendon was trimmed under a microscope to assure optimal accuracy for weight measurement. The muscles were then blotted dry and weighed. Muscles were either frozen in liquid nitrogen‐cooled isopentane for cryosectioning or snap‐frozen in liquid nitrogen for protein extraction. Alternatively, muscles were minced for flow cytometric analysis as described below.

### 6.3 Voluntary wheel running

Mice, housed three per cage, were provided access to a trackable, low‐profile saucer wheels (Med Associates, Inc.) 24 hr per day for 14–28 days to establish consistent running patterns. After establishing baseline, all mice were infected with IAV at an age‐appropriate dose (Figure S1). Wheel rotations were gathered in one‐minute increments via wireless hub and analyzed using wheel manager software (Med Associates, Inc.). Distances travelled per mouse per night (8 p.m.‐6 a.m.) were calculated for each night. Data were collected as the average of three mice per cage for six cages each, and running distance is expressed related to average nightly runs over 10 days pre‐Influenza A.

### 6.4 Immunohistochemistry and fiber size and type assessment

Soleus and EDL serial transverse cryosections (8 μm) were obtained from the Northwestern University Mouse Histology and Phenotyping Laboratory and mounted on glass slides. Sections were fixed in 4% formaldehyde, permeabilized, and blocked. Immunostaining was performed with laminin primary antibody (1:50 dilution; Sigma; Catalog: L9393) followed by Alexa Fluor 568‐conjugated secondary antibody (1:200 dilution; Life Technologies; Catalog: A‐11011). Images were acquired with a Zeiss LSM 510 confocal microscope using a 40x objective (Northwestern University Center for Advanced Microscopy) and analyzed using Zeiss LSM5 Image Browser software. Fiber size was studied by measuring the fibers' minimal inner diameter (at least 100 fibers per muscle), defined as the minimum diameter from inner border to inner border, passing through the center of the muscle fiber. This parameter has been shown to be very insensitive to deviations from the “optimal” cross‐sectioning profile, as compared with direct measure of fiber CSA (Radigan et al., 2019). CSA was calculated using this diameter, and results were expressed as mean CSA ± SE

### 6.5 Bronchoalveolar lavage

Saline fluid (0.8 ml) was lavaged and then aspirated from the lungs of mice through tracheal angiocatheter. Cell counts were measured by trypan blue exclusion and the automated countess system. To measure the cell differential, 200 μl of BAL fluid was placed in cytospin funnels and spun 450 rcf 5 min onto a glass slide using Shandon Cytospin 3 centrifuge. Cells were stained by Wright–Giemsa and manually counted and identified.

### 6.6 IL‐6 measurements

IL‐6 expression assays were performed using a kit from Life technologies (KMC0061C) according to the manufacturer's instructions. Assays were performed on either BAL fluids (acquired as described above) or from 50 μl/well blood plasma drawn from posterior vena cava using a 23‐gauge needle, centrifuged to remove cells, and stored at −80°C. Analysis for each mouse was done in triplicate, and a minimum of five mice were analyzed/condition.

### 6.7 Flow Cytometry and Cell Sorting

Mice were fully perfused through the right ventricle with 20 ml of PBS, and hind limb muscle was removed. Tissue was cut into small pieces with scissors, transferred into C‐tubes (Miltenyi), and processed with a Skeletal Muscle Dissociation Kit according to manufacturer's instructions (Miltenyi). To achieve a single‐cell suspension, two rounds of dissociation were performed using a GentleMACS dissociator (Miltenyi), followed by three rounds of filtration through Falcon 100‐μm, 70‐μm, and then 40‐μm nylon mesh filter units (Thermo Fisher #352340, 352350, and 352360, respectively) into polypropylene 50‐ml Falcon tubes, followed by centrifugation at 300 rcf in an Eppendorf 5810R centrifuge for 15 min. Any remaining red blood cells were lysed by resuspending the pellet in 1ml/tube BD Pharm Lyse (BD Biosciences), and were transferred to flow tubes, pelleted by low‐speed centrifugation 5 min, and resuspended in 0.5ml/tube HBSS containing 0.5 μl/tube viability dye eFluor 506 (eBioscience). Cells were stained in viability dye for 15 min in the dark at room temperature, followed by 2X washes in 1ml MACS buffer (Miltenyi). After live/dead cell staining, pellets are resuspended in 150 μl/tube FcBlock Anti‐mouse CD16/CD32 (Invitrogen) for 30 min in the dark at 4°C to block non‐specific binding. After blocking, cells were divided into three separate tubes at 50 μl each, then stained with a mixture of fluorochrome‐conjugated antibodies in 50 μl/tube, and incubated for an additional 30 min at 4°C in the dark. After antibody incubation, the cells were washed 2X in MACS buffer, and then fixed in 2% paraformaldehyde for 15 min at room temperature in the dark, washed 2X in HBSS, resuspended in 200 μl HBSS, and then stored at 4°C overnight. Data were acquired on a BD LSR Fortessa II flow cytometer using BD FACSDiva software, and compensation and data analyses were performed using FlowJo software (Treestar). Prior to cytometric analysis, 50 μl 123count eBeads (Invitrogen) were added to 200 μl each sample to allow accurate cell counts/gm of tissue. “Fluorescence minus one” controls were used when necessary. Cell populations were identified using sequential gating strategy (Figures 3a,4a,5a). For Foxp3 detection, a staining kit for intracellular antigens was used (eBiosciences). Cells were fixed and permeabilized according to directions, before incubation with Foxp3 antibody prior to analysis. Cell sorting was performed at Northwestern University RLHCCC Flow Cytometry core facility on SORP FACSAria III instrument (BD) with the same optical configuration as LSR II, using a 100‐µm nozzle and 40 psi pressure.

To measure proliferation, EdU was diluted in sterile saline from 100 mg/ml stock in DMSO, and injected intraperitoneally at 2 mg/mouse, 16 hr prior to tissue harvest (Misharin et al., 2017). Single‐cell suspensions were divided into thirds and stained with antibody panels as described in (Figures 3a,4a,5a) with FITC replaced in each panel. After surface antibody staining, cells are fixed and undergo a Click‐IT EdU detection reaction using an Alexa‐Flour 488 picolyl azide and Click‐IT Plus EdU Flow Cytometry Assay Kit (Invitrogen).

### 6.8 Quantitative RT‐PCR

Ten thousand skeletal muscle macrophages were sorted on a FACSAria 6‐Laser Sorter, into 200 μl PicoPure lysis buffer, and stored at −80°C. RNA was harvested following manufacturer's directions for Arcturus PicoPure RNA Isolation Kit (Applied Biosystems). Total RNA was quantified using a NanoDrop 2000c (Thermo Scientific). cDNA was generated using iScript cDNA Synthesis Kit (Bio‐Rad) in a Bio‐Rad T100 thermal cycler, followed by qPCR with SYBR Green (Bio‐Rad) and a Bio‐Rad CFX Connect real‐time system. The following primers were used:

Gapdh (F‐GCA CAG TCA AGG CCG AGA AT, R‐GCC TTC TCC ATG GTG GTG AA)

MerTK (F‐CGT CTG TCC TAA CCG TAC CT, R‐GTA CTG TTG AGG ATA TGG ACT)

Axl (F‐GGT CCC CCT GAG AAC GTT AG, R‐CAT AAG TAC CTC GGG GGT GT)

Tyro3 (F‐AGT GGA ACG GTC TGA TGC TG, R‐AGA ATG GCA CAC CTT CGA CA)

Gas6 (F‐ATG GGT GCA TGA GGA GTT GG, R‐TGT TCG GGT GTA GTT GAG CC)

### 6.9 Western blot analysis

Quadriceps muscle tissue was homogenized with a Tissue Tearor (BioSpec Products, Inc) for 1 min in ice‐cold lysis buffer (Nonidet P‐40 1%, glycerol 10%, NaCl 137 mM, Tris‐HCl pH 7.5 20 mM) containing protease (1 Complete Mini, EDTA‐free tablet, Roche) and phosphatase (sodium fluoride 30 mM, β‐glycerophosphate 250 mM, sodium orthovanadate 1mM) inhibitors. Samples were centrifuged at 9,000 *g*  for 10 min at 4°C, and the supernatant was collected. Protein concentrations were determined with Protein Assay Dye (Bio‐Rad) in a 96‐well plate measured against BSA protein standard dilutions at 595 nm with a Bio‐Rad iMark microplate reader (Bio‐Rad, Hercules CA). Equal amount of protein (40–80 total μg) was loaded on a 9% SDS‐PAGE gel, and Western blot analysis was performed as described previously (Radigan et al., 2019). Incubation with primary antibodies was performed overnight at 4°C. Immunoblots were quantified by densitometry using Image J 1.46r (National Institutes of Health) or Image Studio Software (LI‐COR Inc.). The following antibodies were used: rabbit monoclonal to Fbx32 (Abcam; catalog: ab168372; 1:1,000) or rabbit monoclonal to GAPDH (D16H11) (Cell Signaling; catalog: 5174s; 1:1,000).

### 6.10 Transcriptome profiling via mRNA‐Seq

Ten thousand skeletal muscle macrophages were sorted on a FACSARIA 6‐Laser Sorter, into 200 μl PicoPure lysis buffer, and stored at −80°C. RNA was harvested following manufacturer's directions for Arcturus PicoPure RNA Isolation Kit (Applied Biosystems). RNA quality was assessed with the 4200 TapeStation System (Agilent). Samples with an RNA integrity number (RIN) lower than 7 were discarded. RNA‐seq libraries were prepared from 0.5 ng total RNA using the SMARTer Stranded Total RNA‐Seq Kit v2–Pico Input Mammalian Kit. Libraries were quantified and assessed using the Qubit Fluorimeter (Invitrogen) and 4,200 TapeStation. Libraries were sequenced on NextSeq 500 instrument (Illumina) at 75‐bp length, single‐end reads. The average reading depth across all experiments exceeded 6x10^6^ per sample and over 94% of reads had a Q‐score greater than 30. For RNA‐seq analysis, reads were demultiplexed using bcl2fastq (version 2.17.1.14). Read quality was assessed with FastQC. Low‐quality base calls were trimmed using Trimmomatic (version 0.36). Reads were then aligned to the *Mus musculus* reference genome (with mm10 assembly) using the STAR aligner (version 2.6.0). Counts tables were generated using HTSeq (version 0.6.1). Raw counts were processed in R (version 3.4.4) using edgeR (version 3.20.9) to generate normalized counts. Negative binomial likelihood with default setting followed by generalized linear models fitting was used to estimate differentially expressed genes. FDR q‐values were used to correct for multiple comparisons, and a value of 0.05 was used as a threshold to indicate statistical significance (see online code). Gene Ontology analysis was performed using GOrilla on two unranked gene lists. The RNA‐seq datasets are available at GEO: GSE142580.

### 6.11 In Vitro phagocytosis

After enzymatic digestion, 50 μl of the single‐cell suspensions were incubated with 35 μl of myeloid antibody cocktail with 15 μl/tube Fluoresbrite YG 1.0 micron microspheres (Polysciences Inc., Warrington, PA) that had been opsonized for 30 min at 37°C in by mixing 1 drop beads with 1ml 50% FBS/HBSS, followed by 2X wash/resuspension in HBSS 1 ml (non‐opsonized beads were used as a negative control). Cell suspensions were allowed to uptake beads for 30 min at 37°C in the dark with shaking, followed by two washes in HBSS to remove any unbound beads, and fixation in 2% paraformaldehyde, followed by flow cytometry with a FITC negative myeloid cocktail to detect bead uptake in myeloid cell populations.

## 7 QUANTIFICATION AND STATISTICAL ANALYSIS

### 7.1 Statistical analysis

Differences between groups were determined according to ANOVA. When ANOVA indicated a significant difference, individual differences were examined using *t* tests with a Tukey correction for multiple comparisons, as indicated. All analyses were performed using GraphPad Prism, version 8.4.2 (GraphPad Software, San Diego CA). Data are shown as means ± SEMs. *p* < .05 was considered statistically significant in all tests.

## CONFLICT OF INTEREST

The authors declare no competing financial interests.

## AUTHOR CONTRIBUTIONS

Constance Runyan designed the study, performed experiments, analyzed results, and wrote the manuscript. Lynn C. Welch performed experiments, analyzed results, and wrote the manuscript. Emilia Lecuona, Masahiko Shigemura, Luciano Amarelle, Raul Piseaux‐Aillon, Kinola J.N. Williams, Nikita Joshi, Kiwon Nam, Alexandra C. McQuattie‐Pimentel, Yuliya Politanska, Ziyan Lu, Lango Sichizya, and Satoshi Watanabe performed experiments and analyzed results. Hiam Abdala‐Valencia designed the study, performed experiments, and analyzed results. Nikolay Markov performed bioinformatic analysis and wrote manuscript. GR Scott Budinger designed and supervised the study, wrote manuscript, and provided reagents, resources, and funding. Jacob I Sznajder and Alexander V. Misharin designed and supervised the study, performed analysis, wrote the manuscript, and provided funding for the project. All authors discussed the results and commented on the manuscript.

## Supporting information

Figure S1–S5

Table S1

Table S2

Table S3

Table S4

## Data Availability

The RNA‐seq datasets from this study are openly available at GEO: GSE142580.
