# Supplementary figures and images for: Impaired phagocytic function in CX3CR1+ tissue‐resident skeletal muscle macrophages prevents muscle recovery after influenza A virus‐induced pneumonia in old mice
Source: Aging Cell. 2020 Jul 28;19(9):e13180. doi: 10.1111/acel.13180 (PMC7587460; doi:10.1111/acel.13180)

**A**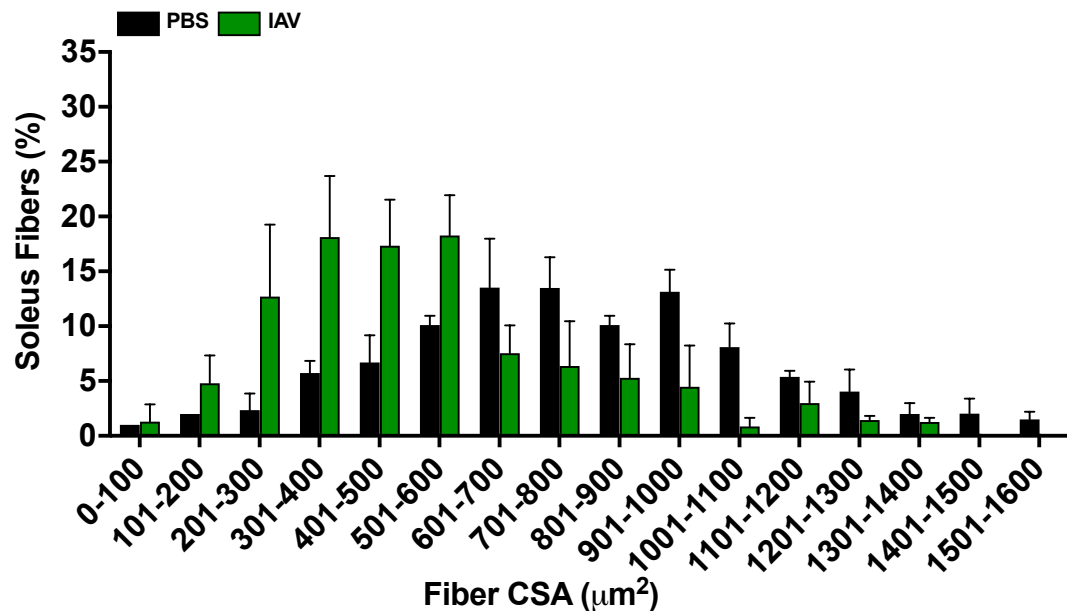**B**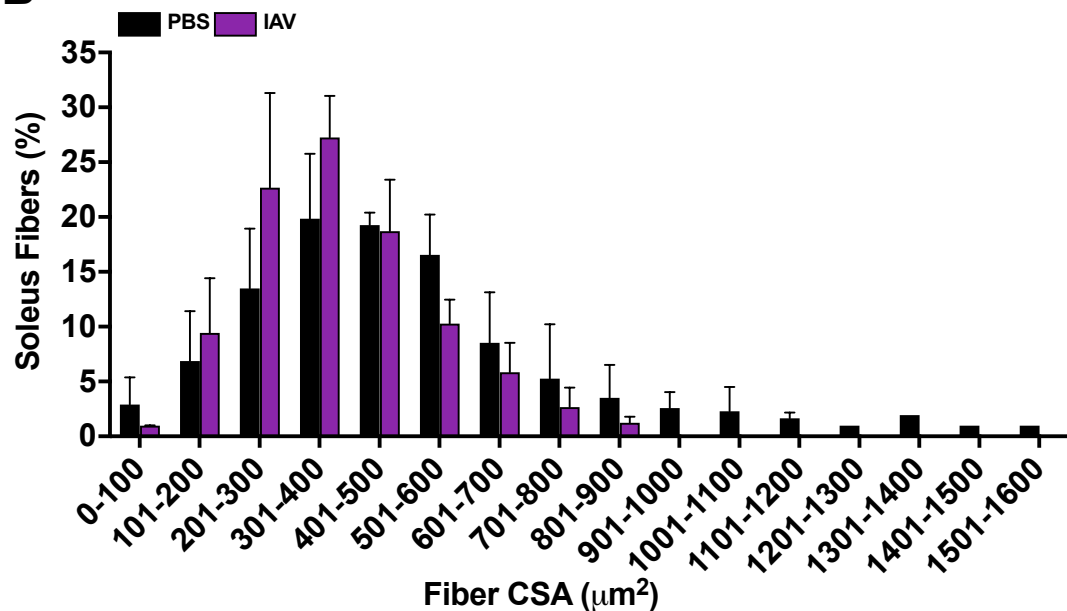

Figure S1

**A**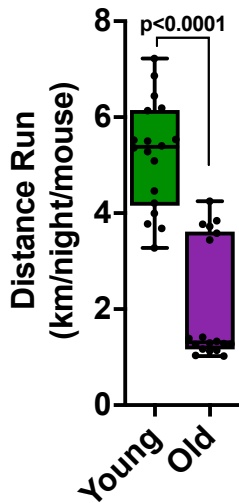**B**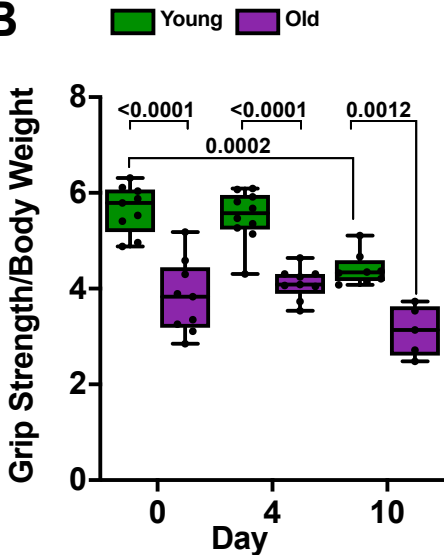

Figure S2

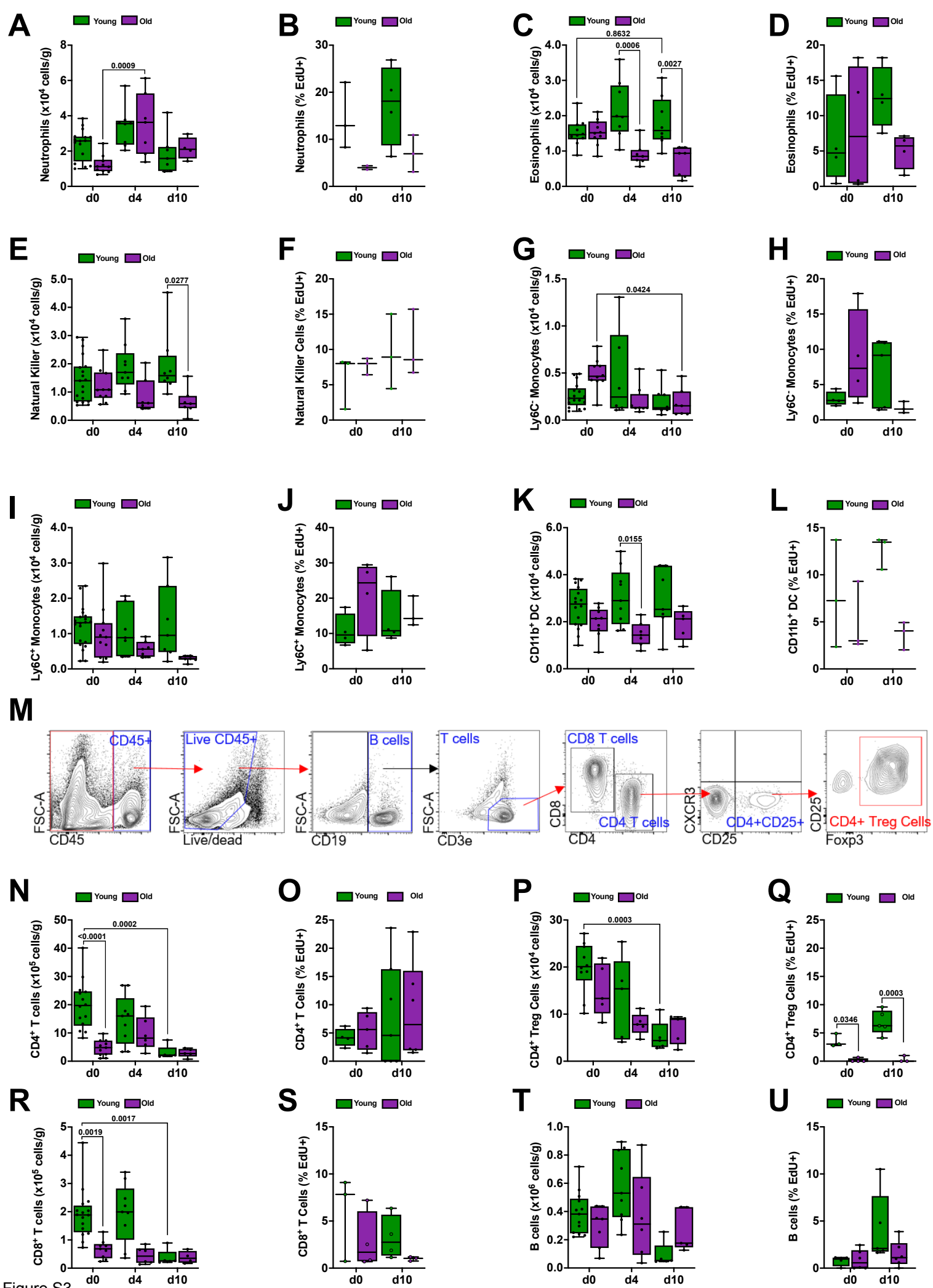

Figure S3

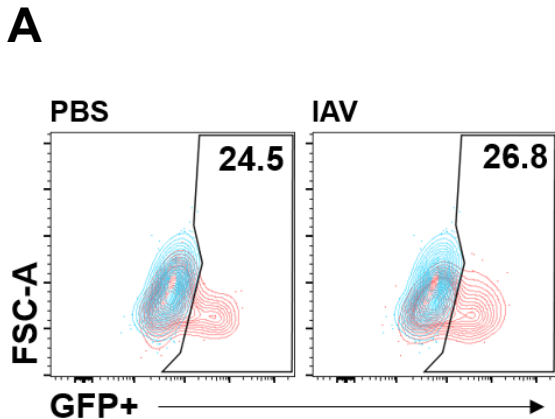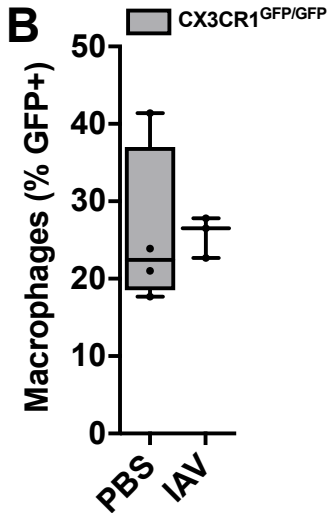

Figure S4

Young WT

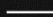

Old WT

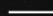

Young Mertk<sup>-/-</sup>

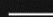

Supplement: Supplementary file 1 — Figure S1–S5 [file ACEL-19-e13180-s001.pdf]
